# Risk of Readmission Among HIV Patients in Public Portuguese Hospitals: Longitudinal Multilevel Population-Based Study

**DOI:** 10.3389/fpubh.2020.00015

**Published:** 2020-02-21

**Authors:** Ahmed N. Shaaban, Sara S. Dias, Zelia Muggli, Bárbara Peleteiro, Maria Rosario O. Martins

**Affiliations:** ^1^Global Health and Tropical Medicine, Institute of Hygiene and Tropical Medicine, NOVA University of Lisboa, Lisbon, Portugal; ^2^EpiDoC Unit – CEDOC, NOVA Medical School – Universidade Nova de Lisboa (NMS-UNL), Lisbon, Portugal; ^3^ciTechCare, Escola Superior de Saúde De Leiria (ESSLei), Instituto Politécnico de Leiria (IPLeiria), Leiria, Portugal; ^4^EPIUnit - Instituto de Saúde Pública, Universidade do Porto, Porto, Portugal; ^5^Departamento de Ciências da Saúde Pública e Forenses e Educação Médica, Faculdade de Medicina da Universidade do Porto, Porto, Portugal

**Keywords:** HIV/AIDS, quality of care (measurement), 30-day readmission, hospital performance indicators, random effects model

## Abstract

**Background:** Thirty-day hospital readmission is receiving growing attention as an indicator of the quality of hospital care. Understanding factors associated with 30-day hospital readmission among HIV patients in Portugal is essential given the high burden cost of HIV hospitalizations in Portugal, a country suffering from financial constrains for almost 10 years.

**Objectives:** We aimed to estimate the 30-day hospital readmission rates among HIV patients in Portugal and to identify its determinants using population-based data for Portuguese public hospitals.

**Study Design:** A multilevel longitudinal population-based study.

**Methods:** Between January 2009 and December 2014, a total of 37,134 registered discharges in the Portuguese National Health Service (NHS) facilities with HIV/AIDS as a main or secondary cause of admission were analyzed. Logistic regression was used to compare 30-day hospital readmission categories by computing odds ratio (OR) and corresponding 95% confidence intervals (95% CIs). A normal random effects model was used to determine unmeasured factors specific to each hospital.

**Results:** A total of 4914 (13.2%, 95% CI: 12.9%−13.6%) hospitalizations had a subsequent 30-day readmission. Hospitalizations that included exit against medical opinion (OR = 1.18, 95% CI: 1.01–1.39), scheduled admissions (OR = 1.71, 95% CI: 1.58–1.85), and tuberculosis infection (OR = 1.20, 95% CI: 1.05–1.38) exhibited a higher risk of hospitalizations with subsequent 30-day readmission. In contrast, hospitalizations that included females (OR = 0.87, 95% CI: 0.81–0.94), a transfer to another facility (OR = 0.78, 95% CI: 0.67–0.91), and having a responsible financial institution (OR = 0.63, 95% CI: 0.55–0.72) exhibited a lower risk of hospitalizations with subsequent 30-day readmission. Hospitalizations associated with higher number of diagnosis, older ages, or hospitalizations during the economic crisis showed an increasing trend of 30-day readmission, whereas an opposite trend was observed for hospitalizations with higher number of procedures. Significant differences exist between hospital quality, adjusting for other factors.

**Conclusion:** This study analyzes the indicators of 30-day hospital readmission among HIV patients in Portugal and provides useful information for enlightening policymakers and health care providers for developing health policies that can reduce costs associated with HIV hospitalizations.

## Introduction

Thirty-day readmission rate has obtained a growing attention as a benchmark indicator for measuring hospital's quality and hospital's performance (1–7). However, although previous studies have recognized hospitalizations with subsequent 30-day readmission as a cost-driving cause and as a cause for frequent problems among people living with HIV (7), there remains a scarcity of research pertaining to the factors associated with readmissions rates, especially when it comes to assessing readmission among HIV patients in Portugal. Admissions among HIV patients still pose considerable challenges to the Portuguese health system. In general, and despite the significant health reforms in Portugal in recent decades (8), HIV infection continues to be a main public health concern (9–11). In Portugal, costs associated with HIV/AIDS hospitalizations represent a substantial economic burden, ranked as the second major diagnosis category (12). In addition, and after the financial crisis that hit the country in 2011, Portugal was obligated to sign up for a bailout program from several funding entities including the European Central Bank and the IMF (13). As a result, the country went through strict fiscal austerity that resulted in budget cuts, reduction of spending on sensitive health sectors, and restructuring numerous public entities including the National AIDS Program (NAP) (12–15). Given these unfavorable economic conditions, and the strains on the Portuguese's health care system with the growing demands on health care resources, it is more important than ever to integrate efficiency measures to maximize the benefits given the available resources. A best-practice readmissions target is fewer 30-day readmission rates, and fewer differences in the care provided among hospitals, without affecting the clinical outcomes for the patients admitted to the hospital and without increasing allocated resources. Since the Portuguese government set the policy of cost reduction as a target to stabilize the economic situation, a considerable work should be devoted to controlling the factors and indicators that tend to push readmission expenses further. Accordingly, this study aims to describe and analyze factors associated with the subsequent 30-day readmission among HIV patients in Portugal by applying ordinary and multilevel logistic regression models to 30-day readmission outcomes in order to compare the two methods with the focus on the interpretation of hospital-level risk factors and to identify differences in hospital quality for the period 2009–2014.

## Methods

### Study Design

We used a longitudinal multilevel population-based study. Data are arranged in a natural hierarchy as in the context of patients-in-hospitals two-level data. Patients within a given hospital typically tend to be more alike than patients across different hospitals in measured and unmeasured characteristics predictive of outcome (16). Accordingly, a multilevel logistic regression model is appropriate to consider the impact of risk factors on readmissions while adjusting for variations in hospitilizations and hospital characteristics (16, 17). Disregarding the clustering present in multilevel data results in an inflated number of independent observations at the hospital level of the hierarchy, and accordingly will underestimate the magnitude of the effect of hospital-level's standard error as occurs in ordinary logistic regression (16). The different empirical models estimated are explained in the *Statistical techniques*.

### Data Collection and Source

The present analysis is based on data collected as part of the national registered discharges among the Portuguese National Health Service (NHS) facilities. We reviewed hospitalization records for all HIV/AIDS patients admitted in Portugal, between 1st January, 2009, and 31st December, 2014, in 48 hospitals in Portugal. These data are anonymous, refer to the DRGs, and were obtained through the Central Health System Administration (ACSS) (18). Each record corresponds to a discharge episode and contains information collected while the patients were admitted to the hospital.

### Study Participants

A total of 37,134 discharges among patients aged 18 years or older were included in the study. For the purpose of this study, data about discharges with HIV/AIDS as a main or secondary cause of admission, [ICD-9, 042, V08], were analyzed. In addition, readmission episodes and the time span between the readmission and the last discharge were calculated. Primary and secondary diagnoses and procedures were coded according to the International Classification of Diseases, 9th Revision, Clinical Modification (ICD-9-CM). Nineteen secondary diagnoses and up to 20 procedures were considered in this study.

### Variables

The main outcome is 30-day readmission. To determine 30-day readmission rate, we used a unique fictional code included in the data that allows determining how many episodes correspond to the same user, in the same institution. This fictional code does not identify the user or allow its identification afterward. Accordingly, readmission episodes and the time span between the readmission and the last discharge were calculated. Our main outcome was created as follows: *Y* = 0 if hospitalizations without subsequent 30-day readmission, *Y* = 1 if hospitalizations with subsequent 30-day readmission(s).

We considered the following independent variables: demographic characteristics (age, sex, insurance), index hospitalization [admission type (urgent or scheduled), type of intervention (surgical or not), diagnoses and procedures (number of diagnoses, number of procedure), associated TB Infection (yes or no)], and prior health care utilization (mode of transfer, destination after discharge). Since several hospitals have been merged in one hospital during the period between 2009 and 2014, we created a dummy variable (hospital merge dummy) to categorize hospitals according to the merging status (Yes: merged, No: Not merged) to be able to study the effect of merging on hospital quality.

### Statistical Analysis

We used the Pearson chi-squared test to compare nominal variables, and the non-parametric tests for ordinal variables. Univariate and multivariate logistic models were estimated to identify the determinants of hospitalizations with subsequent 30-day readmission. Odds ratio (OR) and corresponding 95% confidence intervals (95% CIs) were calculated. For the multilevel approach, a binomial random effects model with a logit link function was used to study the relationship between independent variables and the main outcome. A normal random effect for the hospitals was included and should be interpreted as differences in hospital quality/performance. Multiple comparisons of hospital effects were done by constructing 95% CIs for random effects. All analyses were conducted with STATA®, version 11.2 (StataCorp LP, College Station, Texas, USA), and RStudio, namely the library MASS.

### Statistical Techniques

First, ordinary crude and adjusted logistic regression models (16, 19) were applied to assess the influence of risk factors on 30-day readmission. If we assume that *p*_*ij*_ is the probability and $\frac{p_{ij}}{1-p_{ij}}$ is the odds of readmission for hospitalization *j* in hospital *i*, the specification and the equation of the ordinary logistic model is as follows:

(1) $$\begin{array}{l} \text{log }\left(\frac{p_{ij}}{1-p_{ij}}\right)\;=\;\beta_{0}+\;\overset{R}{\underset{k=1}{\sum }}\;\beta_{k}X_{ijk} \end{array}$$

where the *X*_*ijk*_'s represent a patient's values of *R* risk factors, and β_1_… β_*R*_ are regression coefficients corresponding to each risk factor. For a given risk factor, its coefficient β_*k*_ is the log OR comparing the effect on 30-day readmission of the risk factor's presence with its absence (16), if a risk factor is an indicator, for example, of associated TB infection (1 if yes, 0 if no). Exponentiating $\beta_{k}\;(e^{\beta_{k}}$) gives the corresponding OR. In ordinary logistic regression, hospital characteristics are treated the same as hospitalization-level risk factors (16), and hospitalization independent variable *Y*_*ij*_ is a binomial variable with 30-day readmission probability *p*_*ij*_.

Multilevel logistic regression (16, 20) assumes that each hospital has its own underlying 30-day readmission probability and this varies from hospital to hospital. In this multilevel model, the logistic regression for 30-day readmission includes an additional term, *u*_*i*_, which is the hospital-level random effects as a predictor variable:

(2) $$\begin{array}{l} \text{log }\left(\frac{p_{ij}^{*}}{1-p_{ij}^{*}}\right)=\;u_{i}+\;\beta_{0}+\;\sum_{k=1}^{R}\;\beta_{k}X_{ijk} \end{array}$$

In this model, $p_{ij}^{*}$ is the probability that hospitalization *j* in hospital *i* will be readmitted within 30 days of the last discharge. The probability depends on the value of the random effects, *u*_*i*_, for that hospital (16). *u*_*i*_ is the totality of measured and unmeasured hospital-level variables that predict 30-day readmission and are uncorrelated with the individual and hospital-level predictor variables in the model. Accordingly, *u*_*i*_ represents the combination of omitted hospital-level variables (16).

Variation in 30-day readmission propensity between hospitals is accommodated by assuming a normal distribution for *u*_*i*_with mean zero and variance τ^2^. A hospital with *u*_*i*_ = 1 can be thought of as having “average” (compared to other hospitals in the population) 30-day readmission probability (on the log odds scale). Higher values of τ^2^ indicate greater heterogeneity in 30-day readmission among hospitals included. By incorporating the *u*_*i*_ term in the model as a random effects, the interdependencies among hospitalizations within hospitals are considered (16, 20). Hospital case-mix index (CMI) from B to F had been combined with all hospitals included in our study. CMI reflects the clinical complexity of a particular inpatient population in a medical care environment, and it measures the relative costs and resources needed to treat this inpatient population (21). In our study, hospitals with CMI B are considered receiving patients in less complicated conditions while hospitals with CMI E are considered receiving patients in worst conditions. Group F refers to hospitals treating specific diseases, such as oncological institutes.

We included a prediction model by measuring the area under the Receiver Operating Characteristics curve (AUC–ROC). AUC–ROC curve is a performance measurement for classification problem at various thresholds settings. ROC is a probability curve and AUC represents degree or measure of separability. The curve indicates the ability of the model in distinguishing between classes. In logistic regression, the higher the AUC, the better the model is at discriminating between discharges with and without 30-day readmissions.

## Results

Between 1st January 2009 and 31st December 2014, there were 37,134 registered discharges in the Portuguese NHS facilities with HIV/AIDS as a main or secondary cause of admission. Table 1 presents the univariate analysis and the main charismatics of participants, according to readmission status. A total of 32,220 (86.8%, 95% CI: 86.4−87.1%) of the admissions were hospitalizations without subsequent 30-day readmission(s), while 4914 (13.2%, 95% CI: 12.9−13.6%) were hospitalizations with subsequent 30-day readmission(s). Among the total admissions, 25,060 (67.5%) hospitalizations were recorded among males. The median age was 44 years with interquartile range between 37 and 52 years. Among individuals with subsequent 30-day readmission(s), there was a higher proportion of subjects with no financial institution when compared to subjects without subsequent 30-day readmission, 9.1 and 3.9%, respectively. The urgent admission was the main type of admission in both groups, 70.7 and 61.6%, respectively. Hospitalizations with subsequent 30-day readmission(s) presented the lowest proportion of subjects having surgical interventions (13.7 vs. 24.9%). In addition, the same category presented a higher proportion of exit against medical advice (AMA) in comparison with the other group (4.1 vs. 3.4%).

**Table 1:** Characteristics of the hospitalizations according to the main outcome: *Y* = 0 if hospitalizations without subsequent 30-day readmission, *Y* = 1 if hospitalizations with subsequent 30-day readmission(s).

	**All hospitalizations**		**Hospitalizations without subsequent 30-day readmission**		**Hospitalizations with subsequent 30-day readmission**		***P*-value**
	***N***	**%**	***N***	**%**	***N***	**%**	
**Sex** ***n*** **(%)**							
Men	25,060	67.5	21,485	66.7	3,575	72.8	<0.001
Women	12,074	32.5	10,735	33.3	1,339	27.2	
**Admission type** ***n*** **(%)**							
Urgent	25,818	69.53	22,793	70.74	3,025	61.56	<0.001
Scheduled or others	11,316	30.47	9,427	29.26	1,889	38.44	
**Associated TB infection** ***n*** **(%)**							
No	35,204	94.80	30,579	94.91	4,625	94.12	<0.05
Yes	1,930	5.20	1,641	5.09	289	5.88	
**Mode of transfer** ***n*** **(%)**							
No transfer	33,341	89.79	28,791	89.36	4,550	92.59	<0.001
Transfer to other facility^a^	3,793	10.21	3,429	10.64	364	7.41	
**Responsible financial institution SNS** ***n*** **(%)**							<0.001
No	1,711	4.61	1,262	3.92	449	9.14	
Yes	35,423	95.39	30,958	96.08	4,465	90.86	
**Destination after discharge** ***n*** **(%)**							
Home	30,452	82.01	26,486	82.20	3,966	80.71	<0.001
To another institution with hospitalization	1,793	4.83	1,652	5.13	141	2.87	
Exit against medical advice	1,284	3.46	1,083	3.36	201	4.09	
Deceased	3,230	8.70	2,687	8.34	543	11.05	
Special service^b^	375	1.01	312	0.97	63	1.28	
**Year** ***n*** **(%)**							
2009	6,200	16.70	5,468	16.97	732	14.90	<0.001
2010	6,259	16.86	5,515	17.12	744	15.14	
2011	6,684	18.00	5,743	17.82	941	19.15	
2012	6,715	18.08	5,726	17.77	989	20.13	
2013	6,689	18.01	5,734	17.80	955	19.43	
2014	4,587	12.35	4,034	12.52	553	11.25	
**Age** ***n*** **(%)**							
18–25	938	2.5	855	2.7	83	1.7	<0.001
26–35	6,650	17.9	5,739	17.8	911	18.5	
36–45	13,357	36.0	11,607	36.0	1,753	35.7	
46–55	8,369	22.5	7,256	22.5	1,113	22.7	
≥55	7,820	21.1	6,766	21.0	1,054	21.5	
**Number of diagnosis** ***n*** **(%)**							
Median (IQR)	6 (4-8)		6 (4-8)		6.5 (4-9)		<0.001
< =4	13,203	35.56	11,743	36.45	1,460	29.71	<0.001
5–6	8,653	23.30	7,656	23.76	997	20.29	
7–8	6,202	16.70	5,150	15.98	1,052	21.41	
>8	9,076	24.44	7,671	23.81	1,405	28.59	
**Number of procedure** ***n*** **(%)**							
Median (IQR)	6 (3-9)		6 (3-10)		5 (2-8)		<0.001
< =3	11,615	31.28	9,757	30.28	1,858	37.81	<0.001
4–6	8,950	24.10	7,738	24.02	1,212	24.66	
7–9	7,485	20.16	6,608	20.51	877	17.85	
>9	9,084	24.46	8,117	25.19	967	19.68	
**Type of intervention** ***n*** **(%)**							<0.001
Surgical	8,689	23.40	8,018	24.89	671	13.65	
Medical or others	28,445	76.60	24,202	75.11	4,243	86.35	
**Hospital merge dummy**							0.001
No	23,470	63.20	20,468	63.53	3,002	61.09	
Yes	13,664	36.80	11,752	36.47	1,912	38.91	

*CI, confidence interval; OR, odds ratio; RE, random effects*.

a *Transfer for conducting exams or follow-up or lack of resources or treatment of associated condition*.

b *Special service includes home service, specialized aftercare, palliative care, and long-term hospital care*.

Table 2 presents the ordinary crude and adjusted logistic regression models and the multivariate logistic regression with random effects for the dependent variable *Y* = 1 if readmitted, *Y* = 0 if not. In the random effects model, and in comparison to males, females were less likely to have 30-day readmission episode, holding all other variables constant (OR = 0.87, 95% CI: 0.81–0.94). Scheduled admissions were more likely to be associated with <30-day readmission episode when compared to urgent admissions in the adjusted random effects model (OR = 1.71, 95% CI: 1.58–1.85). Moreover, admissions associated with TB infection were more likely to have subsequent 30-day readmission risk (OR = 1.20, 95% CI: 1.05–1.38) in the adjusted random effects model. Admissions associated with a transfer to another hospital/facility for conducting exams or follow-up or lack of resources or treatment of associated condition were less likely to be readmitted (OR = 0.78, 95% CI: 0.67–0.91) in the ordinary adjusted model. Regarding types of discharge, hospitalizations of patients who exit against medical opinion exhibited a pattern of increasing readmission risk (OR = 1.18, 95% CI: 1.01–1.39) in the random effects model. In addition, admissions associated with patients who deceased later were more likely to be readmitted in the random effects model (OR = 1.47, 95% CI: 1.32–1.64). The opposite is seen in admissions followed by discharged to another institution with hospitalization (OR = 0.61, 95% CI: 0.46–0.80). As regards the year of readmission, admissions in 2011 were more likely to have subsequent 30-day readmission following hospital discharge when compared with 2009 in the crude (OR = 1.22, 95% CI: 1.10–01.36), ordinary adjusted (OR = 1.15, 95% CI: 1.03–1.28), and adjusted with random effects model (OR = 1.13, 95% CI: 1.02–1.27). In addition, admissions occurred in the year 2012 showed the same pattern in the crude (OR = 1.29, 95% CI: 1.16–1.43) and the ordinary adjusted models (OR = 1.14, 95% CI: 1.03–1.27). However, there was a significant decrease in the likelihood of subsequent 30-day readmission in the admissions occurred in 2014 in the ordinary adjusted (OR = 0.70, 95% CI: 0.62–0.80), and the adjusted model with random effects (OR with RE = 0.74, 95% CI: 0.65–0.85). Individuals who have a responsible financial institution (SNS) were less likely to have 30-day readmission (OR = 0.63, 95% CI: 0.55–0.72) in the random effects model. All categories of older ages in the ordinary crude model 26–35 (OR = 1.64, 95% CI: 1.29–2.07), 36–45 (OR = 1.56, 95% CI: 1.23–1.96), 46–55 (OR = 1.58, 95% CI: 1.25–2.00), ≥55 (OR = 1.60, 95% CI: 1.27–2.03) showed a significant increase in the likelihood of readmission risk, when compared to patients aged between 18 and 25 years old. Increasing readmission risk was consistently seen with progressively higher number of diagnosis 5–6 (OR = 1.21, 95% CI: 1.12–1.33), 7–8 (OR = 1.61, 95% CI: 1.46–1.78), >8 (OR = 2.13, 95% CI: 1.92–2.36). The opposite is seen in hospitalizations with progressively higher number of procedures 4–6 (OR = 0.82 95% CI: 0.75–0.90), 7–9 (OR = 0.62, 95% CI: 0.56–0.69), >9 (OR = 0.47, 95% CI: 0.42–0.52), in which higher number of procedures was significantly less likely associated with 30-day readmission risk. Admissions associated with medical interventions were more likely to be followed by a 30-day readmission episode in comparison to admissions associated with surgical interventions (OR = 2.24, 95% CI: 2.034–2.46). The area under ROC curve that reflects the ability of our model to correctly classify discharges with and without 30-day readmission is 0.65 (95% CI: 0.65–0.66).

**Table 2:** Logistic regression for the main outcome: *Y* = 0 if hospitalizations without subsequent 30-day readmission, *Y* = 1 if hospitalizations with subsequent 30-day readmission(s) using GLM with random effects.

	**Constitutional ratio of 30-day readmission % (95% CI)**	**Crude OR (95% CI)**	**Adjusted OR without RE (95% CI)**	**Adjusted OR with RE (95% CI)**
**Sex**				
Men	14.3 (13.8–14.7)	1	1	1
Women	11.1 (10.5–11.7)	0.75 (0.70–0.80)	0.83 (0.78–0.89)	0.87 (0.81–0.94)
**Admission type**				
Urgent	11.7 (11.3–12.1)	1	1	1
Scheduled or others	16.7 (16.0–17.4)	1.51 (1.42–1.61)	1.84 (1.71–1.99)	1.71 (1.58–1.85)
**Associated TB infection**				
No	13.1 (12.8–13.5)	1	1	1
Yes	15.0 (13.4–16.6)	1.16 (1.02–1.32)	1.12 (0.98–1.28)	1.20 (1.05–1.38)
**Mode of transfer**				
No transfer	13.6 (13.3–14.0)	1	1	1
Transfer to other facility^a^	10.0 (8.7–10.5)	0.67 (0.60–0.75)	0.78 (0.67–0.91)	0.98 (0.79–1.20)
**Responsible financial institution SNS**				
No	26.2 (24.2–28.3)	1	1	1
Yes	12.6 (12.3–13.0)	0.40 (0.36–0.45)	0.48 (0.42–0.54)	0.63 (0.55–0.72)
**Destination after discharge**				
Home	13.0 (12.6–13.4)	1	1	1
To another institution with hospitalization	7.9 (6.6–9.1)	0.57 (0.48–0.68)	0.74 (0.59–0.93)	0.61 (0.46–0.80)
Exit against medical advice	15.7 (13.7–17.6)	1.24 (1.06–1.45)	1.14 (0.97–1.34)	1.18 (1.01–1.39)
Deceased	16.8 (15.5–18.1)	1.35 (1.22–1.49)	1.44 (1.30–1.60)	1.47 (1.32–1.64)
Special service^b^	16.8 (13.0–20.6)	1.35 (1.03–1.77)	1.35 (1.02–1.79)	1.23 (0.92–1.64)
**Year**				
2009	11.8 (11.0–12.6)	1	1	1
2010	11.9 (11.1–12.7)	1.00 (0.90–1.12)	0.99 (0.88–1.10)	0.99 (0.88–1.10)
2011	14.1 (13.2–14.9)	1.22 (1.10–1.36)	1.15 (1.03–1.28)	1.13 (1.02–1.27)
2012	14.7 (13.9–15.6)	1.29 (1.16–1.43)	1.14 (1.03–1.27)	1.01 (0.98–1.23)
2013	14.3 (13.4–15.1)	1.24 (1.12–1.38)	1.04 (0.93–1.16)	0.98 (0.88–1.10)
2014	12.1 (11.1–13.0)	1.02 (0.91–1.15)	0.70 (0.62–0.80)	0.74 (0.65–0.85)
**Age**				
18–25	8.8 (7.0–10.7)	1	1	1
26–35	13.7 (12.9–14.5)	1.64 (1.29–2.07)	1.37 (1.07–1.74)	1.26 (0.99–1.61)
36–45	13.1 (12.6–13.7)	1.56 (1.23–1.96)	1.14 (0.90–1.45)	1.09 (0.86–1.39)
46–55	13.3 (12.6–14.0)	1.58 (1.25–2.00)	1.17 (0.92–1.49)	1.17 (0.92–1.49)
≥55	13.5 (12.7–14.2)	1.60 (1.27–2.03)	1.14 (0.90–1.46)	1.16 (0.91–1.48)
**Number of diagnosis**				
<=4	11.1 (10.5–11.6)	1	1	1
5–6	11.5 (10.8–12.2)	1.05 (0.96–1.14)	1.25 (1.14–1.36)	1.21 (1.12–1.33)
7–8	17.0 (16.0–17.9)	1.64 (1.51–1.79)	1.88 (1.72–2.07)	1.61 (1.46–1.78)
>8	15.5 (14.716.2)	1.48 (1.36–1.59)	2.20 (2.00–2.42)	2.13 (1.92–2.36)
**Number of procedure**				
<=3	16.0 (15.3–16.7)	1	1	1
4–6	13.5 (12.8–14.3)	0.82 (0.76–0.89)	0.76 (0.69–0.82)	0.82 (0.75–0.90)
7–9	11.7 (11.0–12.4)	0.70 (0.64–0.76)	0.57 (0.52–0.63)	0.62 (0.56–0.69)
>9	10.6 (10.0–11.3)	0.62 (0.58–0.68)	0.43 (0.39–0.48)	0.47 (0.42–0.52)
**Type of intervention**				
Surgical	7.8 (7.2–8.3)	1	1	1
Medical or others	14.9 (14.5–15.3)	2.09 (1.92–2.28)	2.52 (2.30–2.77)	2.24 (2.03–2.46)
**Hospital merge dummy**				
No	12.8 (12.4–13.2)	1	1	1
Yes	14.0 (13.4–14.6)	1.11 (1.04–1.18)	1.09 (1.02–1.16)	1.17 (0.84–1.65)

*CI, confidence interval; OR, odds ratio; RE, random effects*.

a *Transfer for conducting exams or follow-up or lack of resources or treatment of associated condition*.

b *Special service includes home service, specialized aftercare, palliative care, and long-term hospital care*.

**Figure 1:**
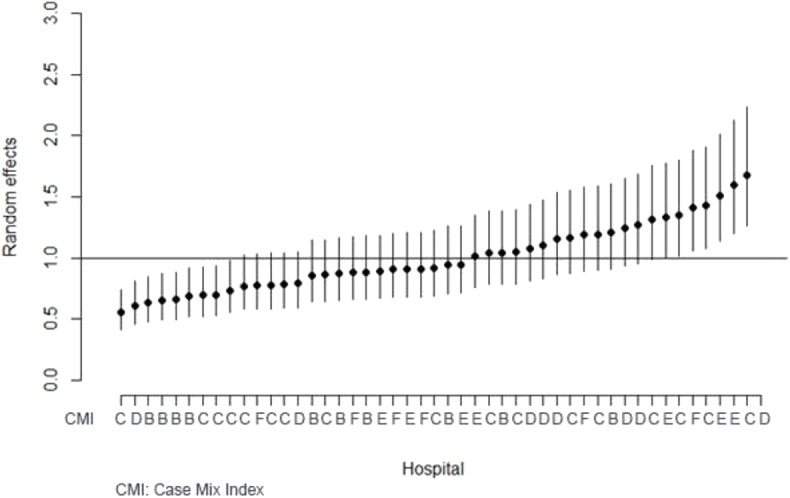
Random effects and their 95% CI for each hospital with case-mix index.

Figure 1 shows the random effects model for 48 hospitals included in the analysis that was used to determine unmeasured and unobserved factors specific to each hospital with their respective 95% CI. The random effects have been conducted as follows: RE < 1 means that the hospital is with more quality when compared to the mean, RE > 1 means that the hospital is with less quality when compared to the mean. The first nine hospitals have random effects and respective 95% CI below 1, being considered with more quality when compared to the mean, while the last seven hospitals' random effects exhibit higher likelihood of readmissions (random effects and corresponding 95% CI above 1). The same figure is combined with hospital's CMI from B to F. The figure shows that hospitals with less quality (more 30-day readmissions) have CMI classified by C, D, E, or F, meaning they receive patients in worse conditions when compared to the rest of the hospitals included in our study, while hospitals with better quality (less 30-day readmissions) present a lower CMI, meaning they receive patients in a less complicated condition: B, C, or D.

## Discussion

The purpose of this study was to estimate the rate of 30-day hospital readmission among HIV patients and to analyze its determinants using the Portuguese national admissions data. The estimated 30-day hospital readmission rate was 13.2% (95% CI: 12.9−13.6%). The results show variability in the patterns of readmission between hospitalizations with and without subsequent 30-day readmission(s). Our study identified increased comorbidity, the absence of a responsible financial institution, age, year of admission and exit against medical opinion as risk factors for of subsequent 30-day readmission. The 30-day readmission rate of 13.2% is almost similar to a study conducted in Brazil (6) and less than the rate of 19.3% reported by a similar study in the United States (1). This lower rate is less surprising if we consider the universal health coverage in Portugal in which all residents have access to health care provided by the NHS (9, 22). Portugal created the NHS to achieve greater equity of access to health care (23).

Our results showed differences in the risk of readmission between years. A significant increase in the likelihood of readmission was noticed after 2009 except for 2014. This finding can be explained by the economic crisis that led to cutting funds to several sectors including health and, as a result, compromising the quality of care (14). In addition, payments to NHS hospitals during the crisis have been reduced, including reduction for payments for in-patient's admissions, day cases, and consultations (24). This reduction was most probably associated with a decrease in hospitals' efficiency and quality by shortening the length of stay, decreasing the number of admissions, or by replacing in-patient admissions by day cases in an attempt to compensate for the effects of the new austerity measures (24). A previous study aimed to analyze the impact of the financial crisis on hospital care use in Portugal on some indicators including the rate of discharges per 100,000 habitants, and the in-patient length of stay (LOS) found the LOS to be significantly shorter with a higher rate of discharges per 100,000 after the crisis onset (24). However, The significant decrease in 30-day readmissions in 2014 is due to the ongoing process of reforming of the hospital sector in Portugal as part of the National Targets for Hospitals Reorganization that included eight initiatives (25). This reforming process started in 2011 in which each hospital should be committed to a 3-year action plan for hospital reorganization with the Regional Health Authority so that reform implementation can be continuously monitored by regional authorities (25). The results of these reforms have been found to positively affect quality and efficiency levels. The outcomes of these reforms have improved several quality measures including a marked reduction in readmissions rates (25).

Adjusted for other variables, admissions for patients not having responsible financial institution were found to increase the possibility of subsequent 30-day readmission risk. These results were in contrast to a previous study in the United States in which individuals who have no insurance were less likely to have 30-day readmission (1). One possible explanation for that could also be the difference in the health insurance system between the two countries. While both uninsured patients in Portugal and the United States have less access to the health services, as regards inpatient and follow-up services, those patients in Portugal can still be admitted to the hospitals through the emergency department without bearing any out-of-pocket payments. In contrast, the association between having no responsible financial institution and lower readmission rate in the United States can be interpreted by the economic barriers to seeking any medical care, including emergency admission, among uninsured patients (1, 26).

Compared to females, males had a higher risk of hospitalizations with subsequent 30-day readmission. When it comes to HIV in Portugal, it is well-known that men carry a disproportionately high burden of the disease. In 2011, the official data show a disproportionate disparity in both the prevalence rates of HIV infection and the mortality rates for deaths by an AIDS-related illness among men in Portugal, 73.6 and 83.9% subsequently (20, 22). In general, this finding can be explained by the fact that men are less likely to seek medical care and to demonstrate suffering or pain (27, 28), which may influence their experiences of living with HIV. In particular, men in Portugal demonstrate more ill health-related conditions throughout their lives as regards alcohol or drug use, unsafe sex, and engagement in risk behaviors than women, which can also be linked to this disproportionate pattern (29). The high rate of subsequent 30-day readmission among males in Portugal requires further investigation and this disparity requires strategies that aim to explain the role of gender and its influence on readmissions among HIV patients in Portugal.

Older ages exhibited patterns of increasing subsequent 30-day readmission risk. Our findings are consistent with previous studies that showed age as a risk factor for morbidity (30, 31), mortality (32, 33), and subsequent 30-day readmission (34) among individuals living with HIV. A possible interpretation for this finding is that age has a significant impact on the pattern of comorbid conditions associated with HIV (35, 36). In addition, and although antiretroviral treatment (ART) had contributed to a substantial increase in life expectancy among individuals living with HIV (37), long-term exposure to these medications may result in antiretroviral toxicity that may contribute to comorbidity (36). It is important to mention that previous studies that examined the interaction between HIV and age showed that some chronic medical conditions are related to antiretroviral toxicity (38, 39). Medicare, which is the federal health insurance program for people who are 65 or older in the United States, found that around 20% of Medicare beneficiaries have subsequent 30-day readmission, and these premature readmissions cost the American public around $15 billion per year (40). This compelling evidence regarding age as a risk for comorbidity and subsequent readmissions demands the inclusion of treatment protocols that guarantee a healthier older age among HIV patients.

Higher number of associated diagnosis exhibited a pattern of increasing readmission risk in our study. A study on the risk factors for all-cause hospital readmission within 30 days of hospital discharge found comorbidity as a strong risk factor for readmission in a general population of patients discharged from the hospital (3). In addition, several studies on the increased 30-day hospital readmission rates, specifically among HIV patients, appointed multiple comorbid chronic conditions as one of the major risk factors for premature readmission (4, 29). This finding supports the evidence that comprehensive care addressing multiple comorbidities is mandatory to decrease readmissions risk among HIV patients.

Leaving hospitalization AMA was also significantly associated with an increase in the rate of 30-day readmission. Leaving hospital AMA will lead to premature discharge, which is most probably associated with multiple readmissions due to uncompleted medical care. This finding is in accordance with previous studies that found a significant positive association between leaving hospital AMA and 30-day readmission (3, 18). Understanding the determinants of leaving the hospital AMA is of paramount importance to be able to identify possible predictors, and potential intervention for AMA, which is a crucial step toward curbing the premature readmissions among HIV patients.

Our results revealed variability in the quality of hospitals included in the random effects model. In other words, this means that, adjusting for other factors, subsequent 30-day readmission is still more represented in some hospitals than others in Portugal. Several facts linked to the geographic inequities in the Portuguese health care system can explain this finding. First, there are remarkable inequities in the allocation of medical human resources and materials in Portugal (41, 42). For example, doctors and human resources are concentrated in Lisbon and Porto, with a lack in the number of doctors in the remote areas (23, 43). Second, patients' socioeconomic characteristics can contribute to this difference in readmissions between hospitals. It is well-known that socioeconomic characteristics are important indicators for health inequalities in Portugal (40, 44). However, some districts in Portugal, namely, the coastal regions, show higher concentrations of younger populations, higher levels of socio-economic indicators, better rates of economic growth, and, as a result, better health status and outcomes (23). In contrast, areas with lower socioeconomic status show poor geographical accessibility (44), and this variation in geographical accessibility affect utilization of health services (45). These findings demand interventions that guarantee health equity and require further analysis of any possible variations in hospital's practices by region that can be associated with higher rates of 30-day readmission.

In addition, our findings revealed that the seven hospitals presenting lower quality are large-scale hospitals that receive large numbers of admissions and with higher bed capacity in comparison to hospitals presenting higher quality. Moreover, the same hospitals have CMI classified by C, D, E, or F, meaning they receive patients in worse conditions when compared to the rest of hospitals included in our study, while hospitals with better quality (less 30-day readmissions) present a lower CMI, meaning they receive patients in a less complicated condition: B, C, or D. These findings warrant further discussions, such as how the CMI can affect the subsequent 30-day readmission.

This study was based on the Portuguese national data for hospital admissions, from which HIV patients were analyzed. Its main strength is providing information on rates and indicators of hospitalizations among HIV patients with subsequent 30-day readmission among all registered admissions in Portugal, resulting in data, which could be used to develop a national health policy to avoid unnecessary readmissions. The aim of this study is in line with the Portuguese national policy of cost reduction and with the recommendations of both the World Health Organization and the European Observatory on Health Systems and Policies which emphasize that “the pursuit of efficiency is one of the central preoccupations of health policymakers and managers, and it is justifiably a cause for such concern” (34). Therefore, reducing hospital readmission rates should be a national priority and health policies should be examined and customized, considering the determinants of readmissions. The main limitation of this study is lacking additional socio-economic factors such as education, income, marital status, and clinical factors to better characterize disease status such as CD4 cell count, HIV RNA <400 copies/ml, and ART at discharge, which are not included in the data collected, and could serve as important indicators for premature readmission. Moreover, number of 30-day readmissions could be underestimated due to death occurring after discharge but within the 30-day period. The subsequent readmission rate of 13.2 in our results assists as a base of comparison for evaluating the quality of inpatient admissions among HIV patients in Portugal. This study offers a unique glimpse for health professionals and policymakers to focus readmission prevention strategies toward patients who are at high risk of readmission including males, elderly, patients who exit AMA, and patients with multiple comorbidities. In particular, it is important to adopt approaches that can address the factors associated with high-risk of readmission and to determine how to decrease any possibly unnecessary readmissions among HIV patients in Portugal.

## Conclusion

We should bear in mind that the shift of HIV infection from a fatal disease into a chronic illness carries substantial challenges to the health system, in terms of hospitalization, readmissions, and the costs associated in the light of limited resources. Findings from our study, supported by a large database to examine 30-day readmission, can help to develop a national health policy to avoid unnecessary readmissions. These findings can set the basis for new insights that can focus on readmission prevention strategies toward patients who are at high risk of readmission, as explained in our study. Moreover, since the study was able to determine differences in hospital quality, this can set the basis for an action plan that can target hospitals with less quality and to further investigate the reason behind lower quality.

## Data Availability Statement

The datasets analyzed in this article are not publicly available. Requests to access the datasets should be directed to http://www.acss.min-saude.pt/.

## Ethics Statement

This study was approved by the Ethics Committee of the Institute of Hygiene and Tropical Medicine (IHMT), Lisbon. This is a secondary analysis of data routinely collected in Portuguese public hospitals, which was conducted under the supervision of the Central Administration of the Health System (ACSS), the legal owner of the DRG database in Portugal. The Ethics Committee of the Institute of Hygiene and Tropical Medicine (IHMT), Lisbon waived the requirement for written informed consent for participants in this study due to the anonymous and confidential nature of the data obtained from the ACSS in which the authors cannot identify any subject on that database, in accordance with the national legislation, the institutional requirements and the Portuguese National Authority of Data Protection for the creation of an individual database.

## Author Contributions

AS performed the formal statistical analysis, interpreted the data, drafted and revised the manuscript. SD, ZM, and BP helped in the interpretation of the data and reviewed the manuscript. SD helped in performing the statistical analysis. MM supervised and helped in performing the statistical analysis, supervised the interpretation of data, reviewed the manuscript, and managed and coordinated responsibility for the research activity planning and execution. AS and MM defined the study hypotheses, conceptualization, and designed the investigation. All authors contributed to the discussion of the result, reviewed the submitted manuscript and approved the manuscript for submission.

### Conflict of Interest

The authors declare that the research was conducted in the absence of any commercial or financial relationships that could be construed as a potential conflict of interest.
